# Efficacy and safety of dinutuximab beta combined with GM-CSF and isotretinoin ± chemotherapy as first-line maintenance treatment for pediatric high-risk neuroblastoma in China

**DOI:** 10.3389/fonc.2026.1765578

**Published:** 2026-02-09

**Authors:** Wen Zhao, Peiyi Yang, Jing Qin, Cheng Huang, Hongjun Fan, Mei Jin, Chao Duan, Ying Wu, Xisi Wang, Chunying Cui, Yan Su

**Affiliations:** 1 Medical Oncology Department, Pediatric Oncology Center, Beijing Children's Hospital, Capital Medical University, National Center for Children's Health, Beijing, China; 2 Laboratory for Clinical Medicine, Capital Medical University, Beijing, China; 3 National Key Clinical Discipline of Pediatric Oncology, Key Laboratory of Major Diseases in Children, Ministry of Education, Beijing, China; 4 School of Pharmaceutical Sciences, Capital Medical University, Beijing, China

**Keywords:** dinutuximab beta, first-line maintenance, GD2- antibody immunotherapy, high-risk neuroblastoma, pediatrics

## Abstract

**Background:**

Real-world evidence on dinutuximab beta for high-risk neuroblastoma (NB) in the Chinese pediatric patients remains limited. This study evaluated clinical efficacy and safety of dinutuximab beta as first-line maintenance therapy in a real-world setting.

**Methods:**

We retrospectively analyzed pediatric patients newly diagnosed with high-risk NB who after induction and consolidation therapy, received dinutuximab beta combined with granulocyte-macrophage colony-stimulating factor and isotretinoin, with or without chemotherapy. The primary outcome was objective response rate (ORR); secondary outcomes included 1- and 2-year event-free survival (EFS) and overall survival (OS) rates, and safety.

**Results:**

Twenty-eight patients were included, with a median age of 56 months (range 27-113) at the initiation of dinutuximab beta. Prior to immunotherapy, 14 patients had achieved complete response (CR) and 14 partial response (PR). Among those with CR, the CR maintenance rate was 78.6% (11/14). In patients with PR, the ORR at the end of treatment (EOT) was 64.3% (9/14). Stratified by treatment modality, PR patients receiving dinutuximab beta with chemotherapy had a higher ORR than those treated with dinutuximab beta alone at EOT [70.0% (7/10) vs. 50.0% (2/4)]. Regarding autologous stem cell transplantation (ASCT) status, all 3 patients who underwent ASCT achieved CR at EOT (ORR: 100%), whereas the ORR among patients without ASCT was 54.5% (6/11). The overall 1-year and 2-year EFS rates were 88.2% [95% confidence interval (CI), 67.4–96.1%)] and 72.4% (95% CI, 41.4–88.8%), respectively; 1-year and 2-year OS rates were both 95.8% (95% CI, 73.9–99.4%). Adverse events (AEs) were common but mostly mild to moderate in severity. Grade 3 or higher AEs occurred predominantly in patients who received combination chemotherapy, including fever, neutropenia, and thrombocytopenia. Pain was effectively managed with most patients requiring only minimal oral morphine (0.01–0.05 mg/kg/d) during cycles 3–5.

**Conclusion:**

Dinutuximab beta shows favorable efficacy for pediatric patients with high-risk NB. Patients treated with prior ASCT or combined with chemotherapy showed trends toward improved response rates and survival outcomes, although optimal treatment regimens required further investigation. AEs are generally manageable, and the use of standardized pain assessment combined with multimodal analgesia has enabled a substantial reduction in morphine exposure.

## 1 Introduction

Neuroblastoma (NB), which originates from the neural crest cells of the sympathetic nervous system, is the most common extracranial solid tumor in children, accounting for 12–15% of cancer-related pediatric mortality (1). In China, the incidence among children aged 0–14 years is 7.72 per million (2). Owing to its aggressive biology and subtle early symptoms, NB is frequently diagnosed at advanced stages (3, 4). Consequently, approximately 50% of patients present with high-risk disease at diagnosis (5), with metastases commonly involving the bone marrow (70–89%) and bone (56–65%) (6–8).

Despite advances in multimodal therapies, including chemotherapy, radiotherapy, surgery, and autologous stem cell transplantation (ASCT), the long-term outcomes for newly diagnosed high-risk NB remain suboptimal, with a 5-year event-free survival (EFS) rate of 37.7% and overall survival (OS) rate of 48.9% (9, 10). Disialoganglioside (GD2) is highly expressed in NB, and GD2-targeted immunotherapies (dinutuximab and dinutuximab beta) selectively bind to GD2 on NB cell membranes, mediating antitumor effects through both antibody-dependent cell-mediated and complement-dependent cytotoxicity (11, 12). The advent of GD2-targeted therapies has reshaped the maintenance phase of the first-line treatment (13–15), and current guidelines recommend dinutuximab or dinutuximab beta as standard therapy (16–18). A landmark study demonstrated that adding dinutuximab and cytokines to isotretinoin improved the 2-year EFS (66% vs. 46%, *p* = 0.01) and OS rates (86% vs. 75%, *p* = 0.02) compared to isotretinoin alone (13). Similarly, dinutuximab beta increased 5-year OS rate from 50% to 64% as first-line maintenance therapy (*p* < 0.0001) (19). Despite these encouraging outcomes, there remains an urgent need to optimize anti-GD2 immunotherapy-based maintenance strategies for enhanced efficacy against newly diagnosed NB.

In China, dinutuximab beta was approved in August 2021 for patients aged ≥12 months with high-risk NB and for those with relapsed or refractory disease. However, real-world evidence in the Chinese population remains limited, constraining clinical decision-making and individualized therapy. This study aims to describe the clinical efficacy and safety of long-term continuous-infusion dinutuximab beta as first-line maintenance therapy, combined with granulocyte-macrophage colony-stimulating factor (GM-CSF) and isotretinoin, with or without chemotherapy, in a real-world setting in China.

## 2 Methods

### 2.1 Study design

This single-center retrospective single-arm observational study included patients with high-risk NB who received dinutuximab beta as first-line maintenance therapy between May 2021 and July 2024 in Beijing Children’s Hospital, Capital Medical University. All patients underwent long-term continuous infusion of dinutuximab beta in combination with subcutaneous GM-CSF, and oral isotretinoin, with or without chemotherapy. The primary outcome was the objective response rate (ORR), and the secondary outcomes included 1- and 2-year EFS and OS rates, and safety. The cutoff date was January 2025.

This study was approved by the Institutional Review Board of Beijing Children’s Hospital (2021-E-029-Y) and was conducted in accordance with the principles of the Declaration of Helsinki. Written informed consent was waived.

### 2.2 Patients

Eligible patients were aged 1–18 years and diagnosed with high-risk NB (stage 4) according to the International Neuroblastoma Risk Group Staging System (20). All patients received induction therapy (chemotherapy and surgery), followed by consolidation treatment with or without ASCT and/or radiotherapy (**Supplementary Tables S1, S2**), in accordance with the Beijing Children’s Hospital NB treatment guidelines (BCH-NB-2019) (10). Inclusion required complete response (CR) or partial response (PR) before initiating dinutuximab beta and at least one post-immunotherapy response assessment. Patients with other primary malignancies were excluded.

### 2.3 Treatment

Patients received five 35-d cycles of dinutuximab beta (100 mg/m^2^ per cycle) administered as a continuous intravenous infusion at a dose of 10 mg/m^2^/d. Two regimens have been used in clinical practice: (1) For patients achieving CR after prior therapies, dinutuximab beta in combination with subcutaneous GM-CSF and oral isotretinoin were administered (regimen 1). (2) For patients achieving PR after prior therapies or those in CR who did not undergo ASCT, dinutuximab beta with GM-CSF, oral isotretinoin, and chemotherapy [either irinotecan/temozolomide (IT) or topotecan/temozolomide (TT)] were administered (regimen 2). In regimen 1, dinutuximab beta was administered on days 4–13, subcutaneous GM−CSF 250 μg/m^2^/d on days 1–14, and oral isotretinoin (160 mg/m^2^/d, divided into two daily doses) on days 16–29. In regimen 2, dinutuximab beta was administered on days 1–10, alongside GM-CSF at 250 ug/m^2^/d subcutaneously on days 6–12, and oral isotretinoin (160 mg/m^2^/d, divided into two daily doses) on days 15–28. The IT regimen comprised irinotecan (50 mg/m^2^/d, intravenously) and temozolomide (100 mg/m^2^/d, orally) on days 1–5; for patients intolerant to IT, the TT regimen substituted topotecan (0.75 mg/m^2^/d, intravenously, days 1–5) with temozolomide dosed as above. Considering the tolerability of combination therapy, dinutuximab beta was not combined with chemotherapy in the first cycle, but was initiated in the second chemotherapy cycle.

Gabapentin and morphine were administered to manage pain. During the first cycle of dinutuximab beta treatment, all patients received intravenous morphine via an infusion pump to prevent and control pain. Morphine dosing was individualized and gradually tapered, transitioning from intravenous to oral administration and subsequent discontinuation.

Glucocorticoids, intravenous immunoglobulins, and investigational agents were not administered during dinutuximab beta treatment. In cases of disease progression or relapse during therapy or follow-up, patients could receive additional antitumor therapy such as chemotherapy or local radiotherapy.

### 2.4 Efficacy and safety assessments

Tumor response was assessed according to the 2017 International Neuroblastoma Response Criteria (21). Efficacy evaluations were performed at baseline (2–4 weeks before immunotherapy treatment initiation), after the 2nd or 3rd cycle, at the end of treatment (EOT), and every 3 months thereafter for up to 3 years. Imaging assessments included meta-iodobenzylguanidine (MIBG) scintigraphy, positron emission tomography/computed tomography (PET/CT), ultrasound, contrast-enhanced cranial magnetic resonance imaging (MRI) or computed tomography (CT) of both primary and metastatic lesions. Bone marrow (BM) was assessed using standard morphological cytology, PHOX2B molecular testing, and flow cytometry. MIBG scintigraphy was evaluated using the Curie score, which semi-quantifies skeletal disease extent within nine body segments on a 0 to 3 scale.

Safety assessments included the documentation of adverse events (AEs), pain severity, vital signs, and laboratory parameters. AEs were graded according to the Common Terminology Criteria for Adverse Events, version 5.0. Pain was measured using age-appropriate scales: the Wong-Baker Faces Pain Rating Scale for patients aged 3–8 years and the Face, Legs, Activity, Cry, Consolability scale for patients younger than 3 years. Opioid dose and duration (intravenous or oral morphine) were also recorded.

### 2.5 Statistical analysis

The ORR was defined as the proportion of patients achieving CR or PR. ORR also were assessed at the subgroup level. EFS was defined as the time from the initiation of dinutuximab beta to progressive disease, relapse, secondary tumor, or death from any cause, whichever event occurred first. OS was defined as the time from the initiation of dinutuximab beta to death from any cause. The 1- and 2-year survival rates [with 95% confidence interval (CI)] were estimated using the Kaplan–Meier method, with group comparisons using the log-rank test. Survival analyses were stratified according to prior response status (CR/PR), MYCN amplification (yes/no), underwent ASCT (yes/no), and treatment regimens (regimen 1/regimen 2).

Descriptive statistics were used to summarize the baseline characteristics, treatments, and safety. Continuous variables were reported as mean, standard deviation, median, and interquartile range; categorical variables were reported as frequencies and percentages. All analyses were performed in SPSS v22.0, with supplementary checks in R where indicated. Two-sided *p* < 0.05 was considered nominally significant.

## 3 Results

### 3.1 Patient characteristics

**Figure 1:**
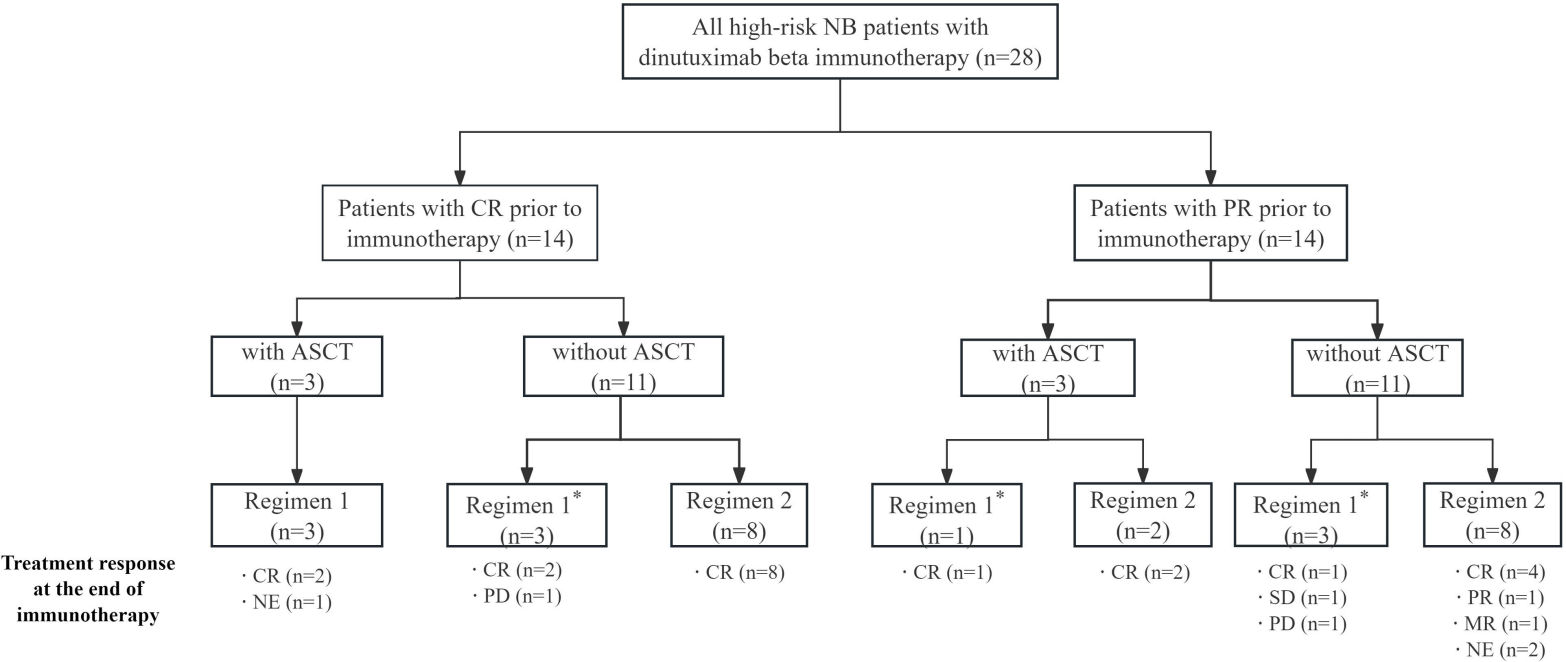
The patients flow. Regimen 1: For patients achieving CR after prior therapies, dinutuximab beta in combination with subcutaneous GM-CSF and oral isotretinoin were administered. Regimen 2: For patients achieving PR after prior therapies or those in CR who did not undergo ASCT, dinutuximab beta with GM-CSF, oral isotretinoin, and chemotherapy (either irinotecan/temozolomide or topotecan/temozolomide) were administered. *Seven patients (three CR without ASCT and four PR) deviated from the protocol and received regimen 1. Because three were ineligible for chemotherapy due to poor organ function, and chemotherapy was avoided in the other four patients-the first cases in the newly introduced dinutuximab beta in China-to minimize risk amid limited clinical experience. ASCT, autologous stem cell transplantation; CR, complete response; GM-CSF, granulocyte-macrophage colony-stimulating factor; NB, neuroblastoma; PR, partial response; NE, not evaluated; MR, minor response; PD, progressive disease; SD, stable disease.

Twenty-eight eligible patients received dinutuximab beta, among them, 14 had achieved CR and 14 PR prior to immunotherapy (**Figure 1**). All three CR patients with prior ASCT received regimen 1 (without chemotherapy). Of the 11 CR patients without ASCT, eight followed the scheduled regimen 2 (with chemotherapy), while three received regimen 1 instead. For the 14 PR patients, regimen 2 was the protocol-specified treatment; however, four patients (one with ASCT and three without ASCT) received regimen 1. Overall, seven patients (three CR without ASCT and four PR) deviated from the treatment protocol. Because three were ineligible for chemotherapy due to poor organ function, and chemotherapy was also avoided in the other four patients - the first cases in the newly introduced dinutuximab beta in China - to minimize risk amid limited clinical experience.

**Table 1** shows that 17 patients (60.7%, 17/28) were male, the median age at diagnosis was 38 months (range 17–101), and that at the initiation of dinutuximab beta was 56 months (range 27–113). MYCN amplification occurred in 10 patients (35.7%). Only six patients (21.4%) underwent ASCT.

**Table 1:** Demographic and clinical characteristics.

Characteristics	Total (N = 28)	Patients with CR^§^ (n=14)	Patients with PR^§^ (n=14)
Gender			
Male	17 (60.7%)	9 (64.3%)	8 (57.1%)
Female	11 (39.3%)	5 (35.7%)	6 (42.9%)
Age at the diagnosis (months), median (range)	38 (17-101)	29.5 (17-78)	43.5 (23-101)
Age at the initiation of dinutuximab beta (months), median (range)	56 (27-113)	40.5 (27-88)	59.5 (33-113)
Primary site			
Retroperitoneal/adrenal glands	24 (85.7%)	12 (85.7%)	12 (85.7%)
Mediastinum	3 (10.7%)	1 (7.1%)	2 (14.3%)
Pelvic cavity	1 (3.6%)	1 (7.1%)	0
MYCN amplification			
Yes	10 (35.7%)	8 (57.1%)	2 (14.3%)
No	17 (60.7%)	5 (35.7%)	12 (85.7%)
Unknown	1 (3.6%)	1 (7.1%)	0
Autologous stem cell transplantation			
Yes	6 (21.4%)	3 (21.4%)	3 (21.4%)
No	22 (78.6%)	11 (78.6%)	11 (78.6%)
Tumor status after induction therapy			
CR	13 (46.4%)	13 (92.9%)	0 (0)
PR	15 (53.6%)	1 (7.1%)	14 (100%)
Residual site before dinutuximab beta treatment^#^			
Bone-only	7 (50.0%)	NA	7 (50.0%)
Bone and bone marrow	2 (14.3%)	NA	2 (14.3%)
Bone and soft tissue	4 (28.6%)	NA	4 (28.6%)
Soft tissue	1 (7.1%)	NA	1 (7.1%)
Chemotherapy cycles before dinutuximab beta, median (range)	9.6 (8-12)	9.0 (7-12)	9.5 (7-15)
Cycles of dinutuximab beta treatment			
6	8 (28.6%)	2 (14.3%)	6 (42.9%)
5	16 (57.1%)	10 (71.4%)	6 (42.9%)
4	1 (3.6%)	1 (7.1%)	0
3	2 (7.1%)	1 (7.1%)	1 (7.1%)
2	1 (3.6%)	0	1 (7.1%)
Dinutuximba beta in combination with chemotherapy			
No	10 (35.7%)	6 (42.9%)	4 (28.6%)
Yes	18 (64.3%)	8 (57.1%)	10 (71.4%)
Irinotecan + Temozolomide	10 (55.6%)	4 (50.0%)	6 (60.0%)
Topotecan + Temozolomide	8 (44.4%)	4 (50.0%)	4 (40.0%)
Receiving DFMO after dinutuximab beta	12 (42.9%)	5 (35.7%)	7 (50.0%)

^§^A total of 28 eligible patients enrolled with 14 achieving CR and 14 PR before immunotherapy. ^#^Percentages are calculated based on 14 patients with residual disease.

CR, complete response; DFMO, difluoromethylornithine; MYCN, v-myc myelocytomatosis viral oncogene; PR, partial response.

At the end of induction therapy, 13 patients (46.4%) achieved CR and 15 (53.6%) achieved PR. One patient with a minimal residual lesion in the right temporal bone (initially evaluated as PR) achieved lesion resolution after radiotherapy and chemotherapy. Before dinutuximab beta initiation, 14 patients (50.0%) had CR and 14 (50.0%) had residual disease (PR). Among the latter, seven (50.0%) had bone-only metastases, four (28.6%) had both bone and soft tissue metastases, two (14.3%) had bone and BM residual disease, and one (7.1%) had soft tissue metastases. Dinutuximab beta was combined with chemotherapy in 18 patients (64.3%), including 8 of CR and 10 of PR.

Compared with patients in CR prior to immunotherapy, those in PR were older at both diagnosis (29.5 vs. 43.5 months) and the initiation of dinutuximab beta (40.5 vs. 59.5 months). MYCN amplification was more frequent in patients with CR prior to dinutuximab beta immunotherapy than in those with PR (57.1% vs. 14.3%).

### 3.2 Tumor response associated with different treatment modalities

Among the patients with CR prior to immunotherapy, 11 (78.6%, 11/14) maintained CR at the last follow-up. The remaining three patients experienced one death from intracranial metastases, one sphenoid bone relapse, and one relapse in the mediastinal and cervical lymph nodes (**Supplementary Table S1**).

Among the 14 patients with residual disease prior to immunotherapy, the ORR was 64.3% (9/14) at EOT and 57.1% (8/14) at the 1-year follow-up (**Table 2**). Stratified by treatment modalities, patients who received dinutuximab beta with chemotherapy had a higher ORR than those who received dinutuximab beta alone [EOT: 70.0% (7/10) vs. 50.0% (2/4); 1-year follow-up: 60.0% (6/10) vs. 50.0% (2/4)]. Based on ASCT status, 11 patients without ASCT had an ORR of 54.5% (6/11), whereas all three patients with ASCT achieved CR at EOT.

**Table 2:** Treatment response in 14 patients with residual disease stratified based on treatment modalities.

Patients, n (%)	Treatment		After treatment	
	Cycle 2 or 3	Cycle 5 or 6 (EOT)	1-year follow-up	Last follow-up
Patients with residual disease (n=14)				
CR	6	8	7	7
PR	2	1	1	2
MR	0	1	0	0
SD	5	1	1	2
PD	1	1	1	3
Not evaluated	0	2	4	0
ORR	57.1%	64.3%	57.1%	64.3%
Dinutuximab beta with chemotherapy (n=10)				
CR	4	6	5	5
PR	2	1	1	2
4MR	0	1	0	0
SD	3	0	0	2
PD	1	0	0	1
Not evaluated	0	2	4	0
ORR	60.0%	70.0%	60.0%	70.0%
Dinutuximab beta without chemotherapy (n=4)				
CR	2	2	2	2
PR	0	0	0	0
MR	0	0	0	0
SD	2	1	1	0
PD	0	1	1	2
ORR	50.0%	50.0%	50.0%	50.0%
PR patients without ASCT (n=11)				
CR	5	5	4	5
PR	1	1	1	2
MR	0	1	0	0
SD	5	1	1	2
PD	0	1	1	2
Not evaluated	0	2	4	0
ORR	54.5%	54.5%	45.5%	63.6%
PR patients with ASCT (n=3)				
CR	1	3	3	2
PR	1	0	0	0
MR	0	0	0	0
SD	0	0	0	0
PD	1	0	0	1
ORR	66.7%	100%	100%	66.7%

CR, complete response; EOT, end of treatment; MR, minor response; ORR, overall response rate; PD, progressive disease; PR, partial response; SD, stable disease.

### 3.3 Tumor response associated with different sites

Among the 13 patients with bone involvement, the ORR was 61.5% (8/13) at EOT and 53.9% (7/13) at 1-year follow-up (**Table 3**). Of the five patients with soft tissue involvement, three achieved CR at EOT and maintained CR until the last follow-up, resulting in an ORR of 60.0% (3/5) at both EOT and 1-year follow-up. Notably, both patients with minimal residual BM disease achieved CR (100%) at cycle 2/3 and maintained CR through the last follow-up.

**Table 3:** Tumor response associated with different metastatic sites in patients with residual disease.

Patients, n (%)	Treatment		After treatment	
	Cycle 2 or 3	Cycle 5 or 6 (EOT)	1-year follow-up	Last follow-up
Bone (n=13)				
CR	5	7	6	6
PR	2	1	1	2
MR	0	1	0	0
SD	5	1	1	2
PD	1	1	1	3
Not evaluated	0	2	4	0
ORR	53.9%	61.5%	53.9%	61.6%
Soft tissue (n=5)				
CR	2	3	3	3
PR	1	0	0	0
MR	0	0	0	0
SD	2	1	1	1
PD	0	0	0	1
Not evaluated	0	1	1	0
ORR	60.0%	60.0%	60.0%	60.0%
BM minimal residual disease (n=2)				
CR	2	2	2	2
ORR	100%	100%	100%	100%

BM, bone marrow; CR, complete response; EOT, end of treatment; MR, minor response; ORR, overall response rate; PD, progressive disease; PR, partial response; SD, stable disease.

### 3.4 MIBG score

**Figure 2:**
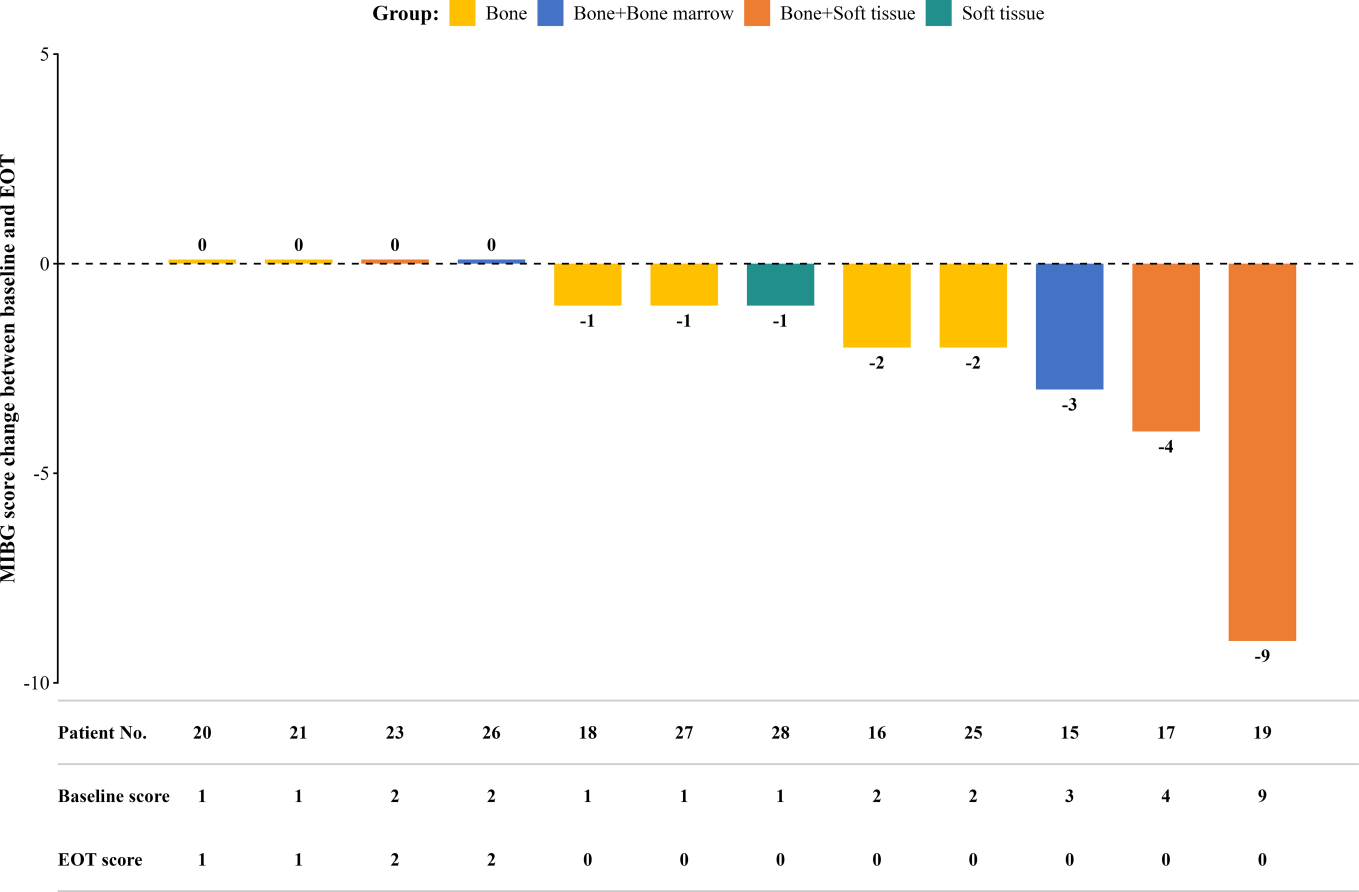
MIBG score of 12 patients before and after treatment. *MIBG, meta-iodobenzylguanidine; EOT, end of treatment*.

Among patients with residual disease who underwent MIBG scans (n =12), the mean baseline and EOT MIBG scores were 2.4 (range 1–9) and 0.5 (range 0–2), respectively with a mean reduction of –1.9 (range –9 to 0). Individual MIBG scores are shown in **Figure 2**.

Lesion response was achieved by 66.7% of the patients (8/12) at EOT, who remained disease-free at the 1-year follow-up (Patients 15, 16, 17, 18, 19, 25, 27, and 28, **Supplementary Table S3**). Patient No. 15 achieved CR at EOT and 1-year follow-up but subsequently relapsed. Four patients had persistent lesions throughout the final follow-up period (Patients 20, 21, 23, and 26).

### 3.5 Survival analyses

**Figure 3:**
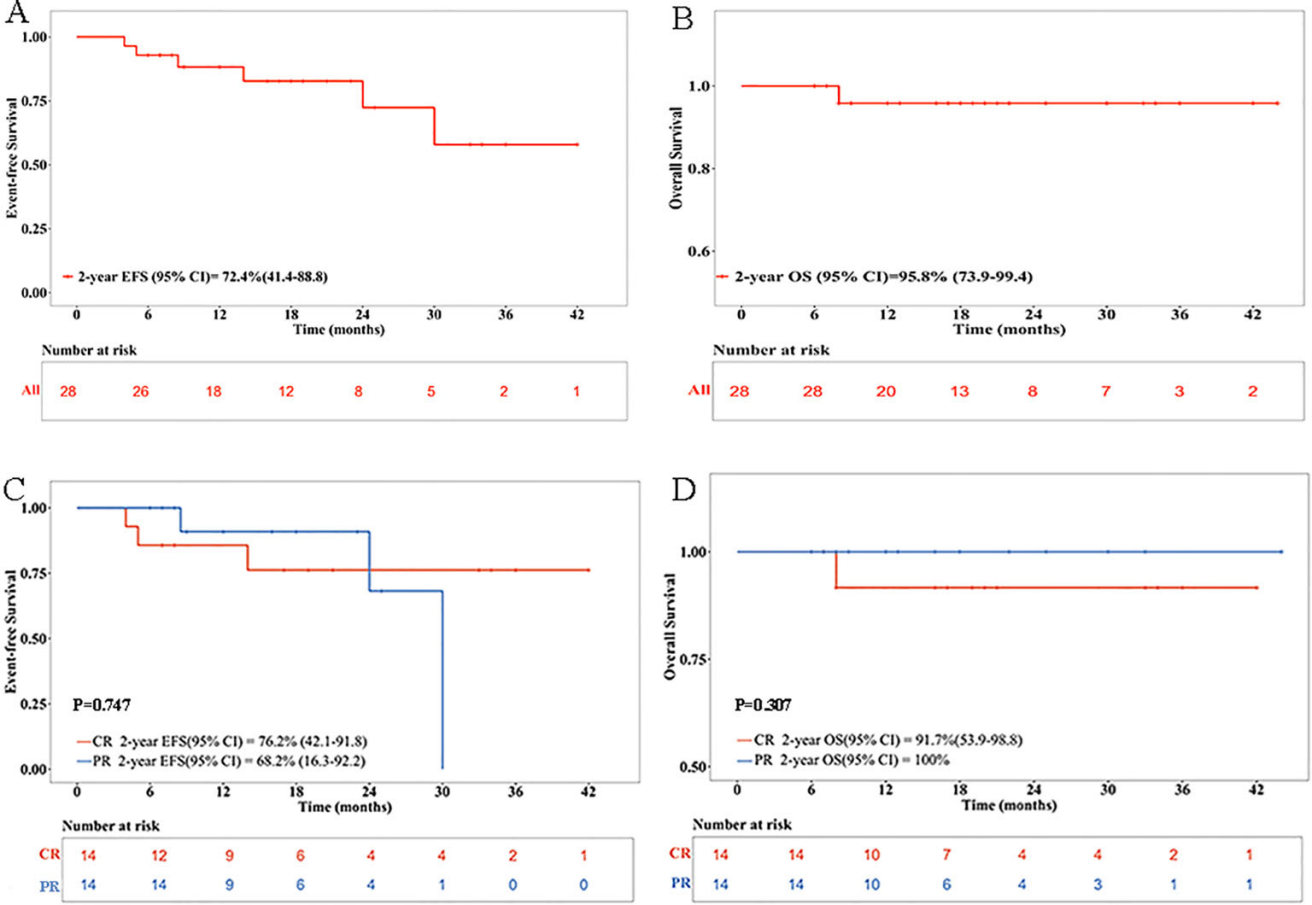
Kaplan–Meier EFS and OS estimates for patients treated with dinutuximab beta. **(A)** EFS rate of all patients. **(B)** OS rate of all patients. **(C)** EFS rate of patients with CR or PR prior to immunotherapy. **(D)** OS rate of patients with CR or PR prior to immunotherapy. CR, complete response; EFS, event-free survival; OS, overall survival; PR, partial response.

At a median follow-up of 19 months (range 6–44), the estimated 1- and 2-year EFS rates for the entire cohort were 88.2% (95% CI, 67.4–96.1%) and 72.4% (95% CI, 41.4–88.8%), respectively; the 1- and 2-year OS rates were both 95.8% (95% CI, 73.9–99.4%) (**Figures 3A, B**).

In the subgroup analysis, patients in prior CR (CR subgroup) showed a lower 1-year EFS rate than those in the PR subgroup [85.7% (95% CI, 53.9–96.2%) vs. 90.9% (95% CI, 50.8–98.7%), *p* = 0.684], but a higher 2-year EFS rate [76.2% (95% CI, 42.1–91.8%) vs. 68.2% (95% CI, 16.3–92.2%), *p* = 0.747]. One- and 2-year OS rates were lower in the CR subgroup than in the PR subgroup [91.7% (95% CI, 53.9–98.8%) vs. 100%, *p* = 0.307; **Figures 3C, D**].

**Table 4** shows that patients with MYCN amplification had lower 2-year EFS rate [70.0% (95% CI, 22.5–91.8%) vs. 80.8% (95% CI, 50.5–93.6%), *p* = 0.620] but numerically higher 2-year OS rate [100% vs. 93.3% (95% CI, 61.3–99.0%), *p* = 0.437] than those without amplification. Patients who underwent ASCT showed numerically better 2-year EFS and OS rates than those who did not [EFS: 83.3% (95% CI 27.3–97.5%) vs. 68.8% (95% CI, 31.6–88.6%), *p* = 0.498; OS: 100% vs. 94.7% (95% CI, 68.1–99.2%), *p* = 0.602]. Patients receiving dinutuximab beta with chemotherapy were inclined to achieved better 2-year EFS [90.9% (95% CI, 50.8–98.7%) vs. 51.4% (95% CI, 14.3–79.6%), *p* = 0.130] and OS rates [100% vs. 88.9% (95% CI, 43.3–98.4%), *p* = 0.185] compared to those receiving dinutuximab beta alone. Additionally, patients treated with DFMO following dinutuximab beta had numerically lower 2-year EFS rate [67.7% (95% CI, 41.2-100.0%) vs. 81.7 (95% CI, 60.8-100.0%), *p* = 0.524] but higher 2-year OS rate [100.0% vs. 90.9% (95% CI, 75.4-100.0%), *p* = 0.317] than those who did not receive DFMO.

**Table 4:** The estimates of survival rates in subgroup.

Subgroups	EFS rate (95% CI)			OS rate (95% CI)		
	1-year	2-year	P-value	1-year	2-year	P-value
MYCN amplification^*^						
Yes (n=10)	100%	70.0% (22.5-91.8%)	0.620	100%	100%	0.437
No (n=17)	80.8% (50.5-93.6%)	80.8% (50.5-93.6%)		93.3% (61.3-99.0%)	93.3% (61.3-99.0%)	
Underwent ASCT						
Yes (n=6)	83.3% (27.3-97.5%)	83.3% (27.3-97.5%)	0.498	100%	100%	0.602
No (n=22)	89.5% (63.6-97.3%)	68.8% (31.6-88.6%)		94.7% (68.1-99.2%)	94.7% (68.1-99.2%)	
Treatment therapy						
Regimen 1-without chemotherapy (n=10)	68.6% (30.5-88.7%)	51.4% (14.3-79.6%)	0.130	88.9% (43.3-98.4%)	88.9% (43.3-98.4%)	0.185
Regimen 2-with chemotherapy (n=18)	100%	90.9% (50.8-98.7%)		100%	100%	
Receiving DFMO						
Yes (n=13)	92.3% (78.9-100.0%)	67.7% (41.2-100.0%)	0.524	100.0%	100.0%	0.317
No (n=15)	81.7% (60.8-100.0%)	81.7% (60.8-100.0%)		90.9% (75.4-100.0%)	90.9% (75.4-100.0%)	

^*^The MYCN status of one patient was unknown.

ASCT, autologous stem cell transplantation; CI, confidence intervals; DFMO, difluoromethylornithine; EFS, event-free survival; MYCN, v-myc myelocytomatosis viral oncogene; OS, overall survival.

### 3.6 Safety

**Figure 4:**
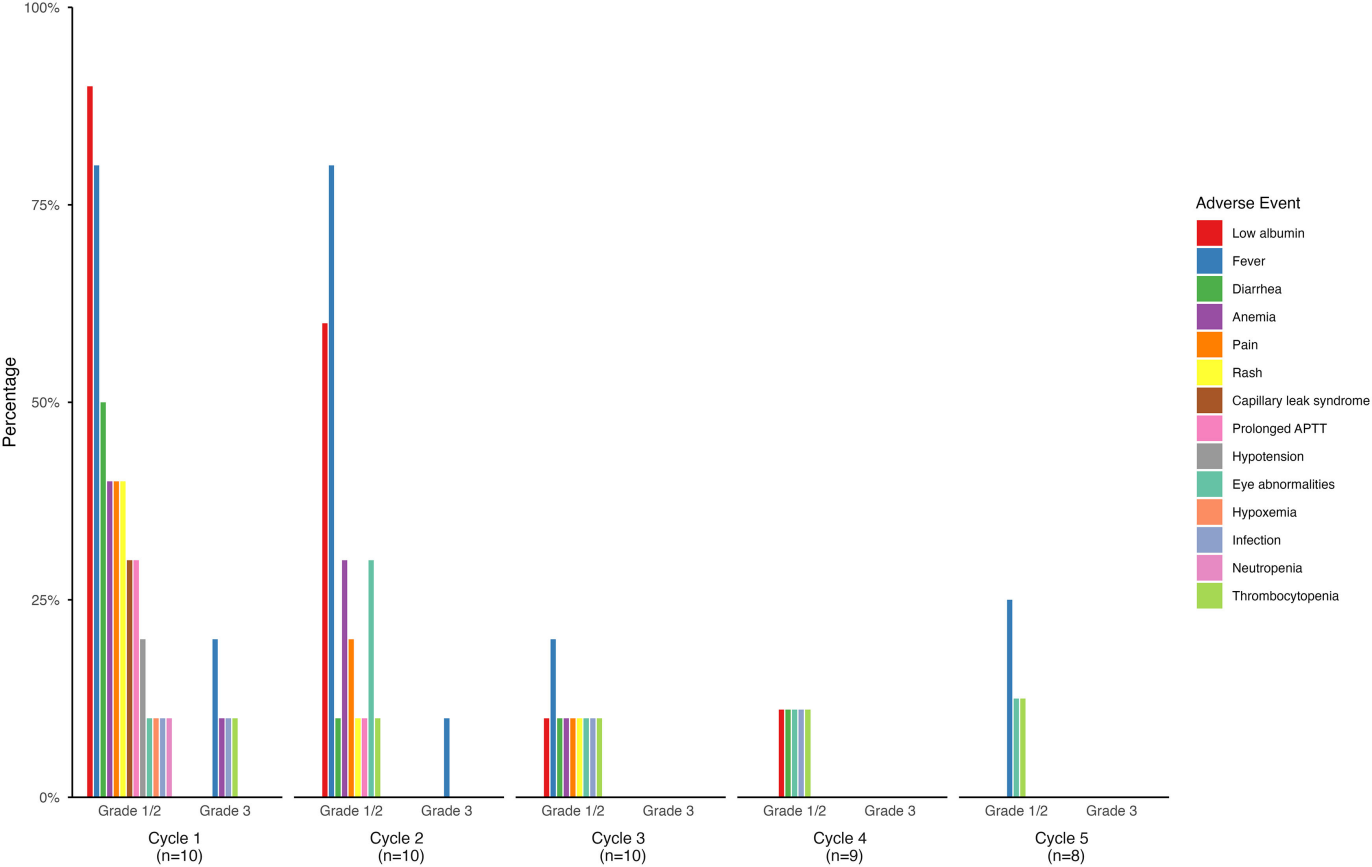
Adverse events in 10 patients who received dinutuximab beta without chemotherapy.

**Figure 5:**
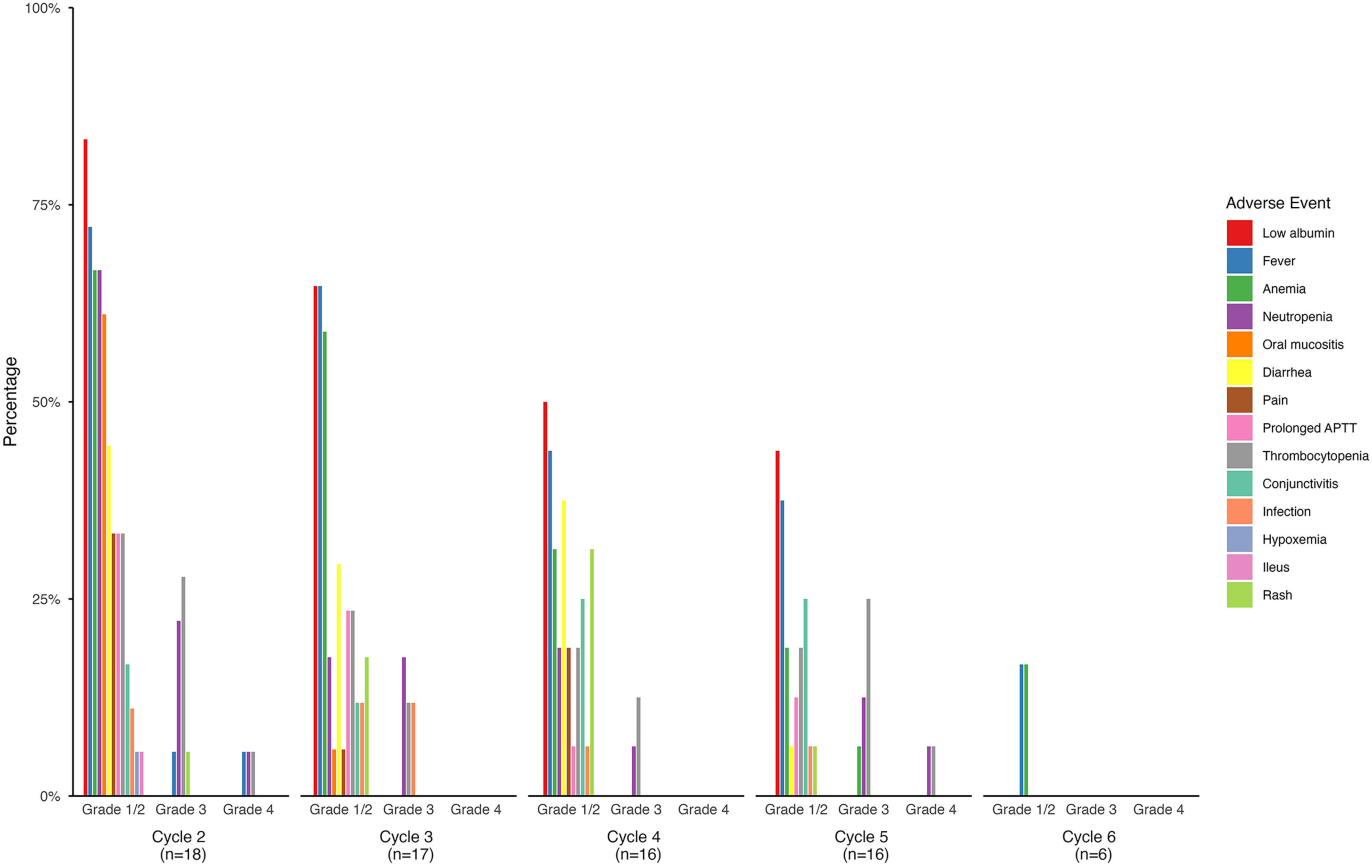
Adverse events in 18 patients who received dinutuximab beta with chemotherapy from cycle 2 to 6. Cycle 2 is the first administration cycle of dinutuximab beta combined with chemotherapy.

All patients experienced AEs, which were most frequent and severe during the first treatment cycle and decreased in subsequent cycles (**Figures 4**, **5**). Most AEs were of grade 1 or 2, and grade 3 or 4 events occurred predominantly in patients who received combination chemotherapy.

In patients receiving dinutuximab beta without chemotherapy (n = 10), common grade 1 or 2 AEs included low albumin (90%), fever (80%), diarrhea (50%), chills (50%), and elevated g-glutamyl transferase (GGT, 50%, **Figure 4**) in cycle 1. Grade 3 events included fever (20%), elevated GGT levels (20%), infection (10%), anemia (10%), thrombocytopenia (10%), and elevated alanine aminotransferase (10%). Fever, eye abnormalities, and thrombocytopenia persisted across five cycles (**Supplementary Table S4**).

Patients receiving dinutuximab beta with chemotherapy (n = 18) experienced more frequent and severe AEs, particularly hematological toxicities (**Figure 5**). In the first administration cycle combined with chemotherapy (cycle 2), the common grade 1 or 2 AEs included fever (72.2%), low albumin (83.3%), anemia (66.7%), and neutropenia (66.7%). Grade 3 neutropenia occurred in 22% of patients and persisted in later cycles. Occasional grade 4 events including fever, neutropenia, and thrombocytopenia were reported. Non-hematological AEs were mostly mild to moderate and included pain, diarrhea, and oral mucositis (**Supplementary Table S5**). Despite the higher AE burden associated with combination chemotherapy, all events were manageable with supportive care, and no treatment-related deaths occurred.

**Figure 6:**
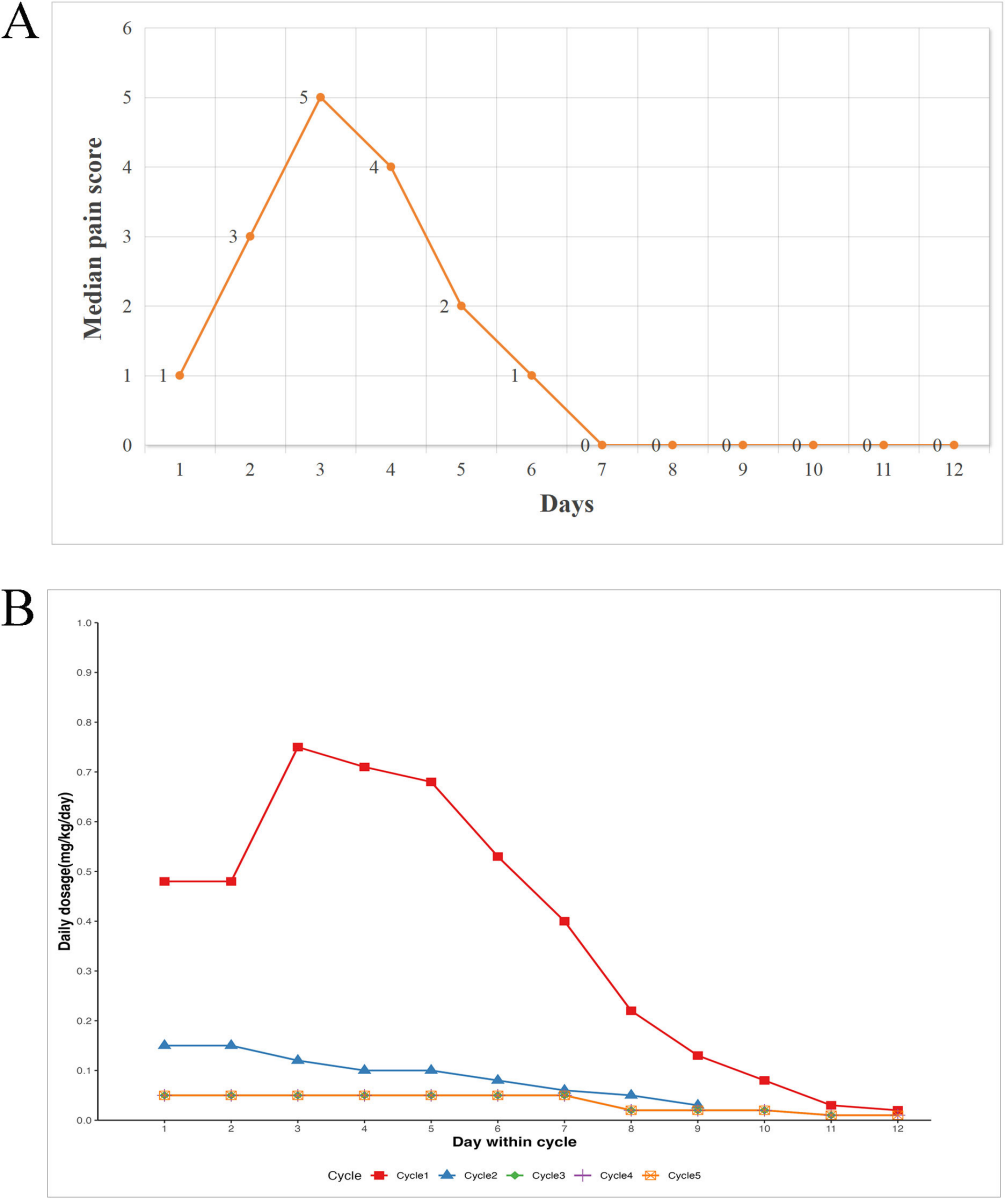
Pain assessment and multimodal analgesia throughout treatment cycles. **(A)** Median pain score in the first cycle. **(B)** Mean dosage of intravenous or oral morphine per cycle.

Pain was the most common dinutuximab beta-related AE. The median pain scores in cycle 1 and mean daily morphine doses across all cycles are shown in **Figure 6**. Intravenous morphine peaked at 0.75 mg/kg/d on day 3 of cycle 1, corresponding to the highest median pain score of 5. By day 4, half of the patients switched to oral morphine, which tapered to 0.05 mg/kg/d by day 8. Morphine requirements in cycles 3–5 were minimal, with low oral doses of 0.01–0.05 mg/kg/d. Pain was effectively controlled with a combination of gabapentin and morphine, allowing all patients to complete the treatment without discontinuation owing to pain.

## 4 Discussion

Despite intensive induction and consolidation regimens, the outcomes of high-risk NB remain challenging. The introduction of GD2-antibody immunotherapy has notably improved maintenance treatment, as evidenced by pivotal trials such as SIOPEN/HR-NBL1 (14, 19) and COG ANBL0032 (13), establishing it as the recommended treatment for high-risk NB (16). This retrospective study evaluated the efficacy and safety of dinutuximab beta as first-line maintenance therapy in Chinese pediatric patients with high-risk NB in a real-world clinical setting. The 2-year EFS and OS rates of the entire cohort were 72.4% (95% CI, 41.4–88.8%) and 95.8% (95% CI, 73.9–99.4%), respectively. The safety of dinutuximab beta was manageable, and no treatment-related deaths occurred.

The study found that 78.6% of the patients with CR prior to immunotherapy maintained in CR at the last follow-up, whereas the ORR was only 64.3% at EOT among those with residual disease. These results align with Yu et al.’s findings (22), who reported a CR maintenance rate of 77.8% and ORR of 63.0% in patients with residual disease. Additionally, Yu et al. reported 2-year EFS and OS rates of 80.1% (95% CI, 66.2-88.8%) and 97.6% (95% CI, 84.3-99.7%) for high-risk NB patients receiving dinutuximab beta as first-line maintenance immunotherapy (22), which were comparable to this study. However, the 1- and 2-year OS rates appeared numerically lower in the CR subgroup compared to the PR subgroup in this study. This difference may be attributed to variations in treatment exposure. Notably, a higher proportion of PR patients (42.9%) completed six cycles compared to CR patients (14.3%). Furthermore, PR patients were more frequently treated with chemotherapy-containing regimens. Although the drug prescribing information recommends a fixed cycle, real-world clinical practice often involves extending treatment beyond five cycles for patients with residual disease, the treatment strategy supported by other studies as potentially beneficial (23, 24). Consequently, the differential treatment exposure likely contributed to the observed survival outcomes between subgroups.

In this study, the addition of dinutuximab beta to chemotherapy resulted in improved ORR at the EOT (with vs. without chemotherapy: 70.0% vs. 50%) and showed a trend toward better survival outcomes in high-risk NB (2-year EFS: 90.9% vs. 51.4%; 2-year OS: 100% vs. 88.9%). The combination of dinutuximab beta and chemotherapy has been widely applied in the treatment of relapsed or refractory (R/R) patients (24, 25). Furthermore, the recent ITCC-SIOPEN BEACON Immuno phase II study, a randomized controlled study, reported superior outcomes with the chemoimmunotherapy combination compared to chemotherapy alone, with ORR of 30.2% versus 18.2% and median progression-free survival (PFS) of 11.1 versus 3.8 months (26). Correspondingly, Chinese expert consensus endorses chemoimmunotherapy for newly diagnosed patients who have not achieved CR prior to immunotherapy or for R/R patients (27). Overall, chemoimmunotherapy has the potential to improve response rates and delay disease progression.

ASCT is a crucial consolidation therapy in the multimodal management of high-risk NB. In this study, 78.6% of patients did not undergo ASCT and instead received dinutuximab beta combined with chemotherapy. After treatment, we observed a trend toward better outcomes in patients with prior ASCT compared to those without ASCT: ORR at EOT were 100% versus 54.5%, and the 2-year EFS rate were 83.3% versus 68.8%, while 2-year OS rates were comparable (100% vs. 94.7%). A Meta-analysis indicated that in patients treated with anti-GD2 immunotherapy, omission ASCT was associated with increased relapse risk (28). Accordingly, ASCT remains recommended by both the NCCN guideline and Chinese expert consensus (16, 18). Therefore, a standardized treatment strategy including ASCT is advised for newly diagnosed high-risk NB patients.

Response rates varied according to the metastatic site. The ORR was 61.5% in patients with bone metastases, 60.6% in those with soft tissue metastases, and 100.0% in those with minimal residual disease limited to the BM in this study. This site-specific pattern was presented by another Chinese study, which reported ORRs of 63.0% for bone metastases and 66.7% for BM involvement with dinutuximab beta combined with chemotherapy (22). These observed response patterns highlight the importance of tailoring the treatment and risk stratification according to metastatic distribution. The bone and BM are the most common metastatic sites in NB, that predicts poor outcomes and remains a major therapeutic challenge (29). This and previous studies indicated that dinutuximab beta combined with chemotherapy may be effective for bone and/or BM disease in high-risk NB; however, whether this advantage translates into a definitive long-term survival benefit requiring further investigation.

The safety profile of dinutuximab beta in this present cohort was manageable, with most AEs being grades 1–2 and grade 3–4 toxicities primarily observed in patients receiving combination chemotherapy. The most common AEs, hypoalbuminemia, fever, and diarrhea, were consistent with previous reports (14, 19). Most AEs occurred during the first or second cycles and declined thereafter. Importantly, pain, a hallmark of AE associated with anti-GD2 antibodies, was mild in our study. While previous studies have reported that the incidence of grade 3–4 pain accounts for 16–26% during dinutuximab beta immunotherapy (14, 19), only grade 1–2 pain was observed in our study. This was attributed to the implementation of a comprehensive pain management strategy including detailed pain assessments, individualized prevention plans, and analgesia, primarily with morphine and gabapentin. None of the patients discontinued antibody therapy because of pain. Only two patients required prolonged opioid use after antibody treatment because of concerns regarding morphine-related toxicity; their opioids were gradually tapered and discontinued after antibody therapy. Notably, the initial morphine infusion rate used in some patients (0.015–0.02 mg/kg/h) and the total morphine exposure in this study were lower than the levels recommended in European guidelines (30) and the drug prescribing information (31). From cycle 2 onward, all 24 patients required only prophylactic oral gabapentin without morphine use, demonstrating the opioid-sparing potential effect of the regimen.

This was a retrospective, single-center study, which may introduce selection bias and limit the generalizability of the results. The small sample size restricts the statistical power, especially for subgroup analyses, potentially affecting the stability of the conclusions. Additionally, the follow-up period was relatively short for a comprehensive assessment of long-term survival and late toxicity.

Dinutuximab beta demonstrates favorable efficacy and manageable safety as first-line maintenance immunotherapy for pediatric patients with high-risk NB. Its combination with chemotherapy shows promising activity in patients who failed to achieve CR or achieved CR without prior ASCT. ASCT also remains a key consolidation strategy to optimize outcomes. AEs are generally manageable, and the use of standardized pain assessment combined with multimodal analgesia has enabled a substantial reduction in morphine exposure. When integrated with optimized supportive care protocols, dinutuximab beta represents a meaningful advancement in the treatment of high-risk NB.

## Data Availability

The raw data supporting the conclusions of this article will be made available by the authors, without undue reservation.
