# Supplementary material for: Efficacy and safety of dinutuximab beta combined with GM-CSF and isotretinoin ± chemotherapy as first-line maintenance treatment for pediatric high-risk neuroblastoma in China
Source: Front Oncol. 2026 Feb 9;16:1765578. doi: 10.3389/fonc.2026.1765578 (PMC12926132; doi:10.3389/fonc.2026.1765578)
Supplement: Supplementary file 1 [file Table1.docx]

Supplementary Material

**Supplementary Table 1. Detail information of 14 patients with CR prior to dinutuximab beta**

| **No.** | **Sex** | **MYCN amplification** | **Cycles of induction chemotherapy** | **Disease status at the end of induction therapy** | **ASCT** | **RT** | **Cycles of dinutuximab beta** | **Combination chemotherapy** | **Other treatments after immunotherapy** | **Follow-up duration (months)** | **Time of event onset (months)** | **Disease status** |
| --- | --- | --- | --- | --- | --- | --- | --- | --- | --- | --- | --- | --- |
| 1 | Male | Yes | 8 | CR | Yes | Yes | 5 | No | No | 36 | - | CR |
| 2 | Female | Yes | 12 | CR | No | Yes | 5 | No | No | 34 | - | CR |
| 3 | Female | Yes | 8 | CR | No | Yes | 6 | Yes/IT×5 | DMFO (14 months after discontinuation of immunotherapy) | 33 | - | CR |
| 4 | Male | No | 8 | CR | No | Yes | 5 | Yes/TT×3 | DMFO (6 months after discontinuation of immunotherapy) | 20 | - | CR |
| 5 | Male | Yes | 10 | CR | No | Yes | 5 | Yes/TT×3 | DMFO (2months after discontinuation of immunotherapy) | 19 | - | CR |
| 6 | Female | No | 8 | CR | No | Yes | 5 | Yes/IT×4 | DMFO (6 months after discontinuation of immunotherapy) | 17 | - | CR |
| 7 | Male | Yes | 10 | CR | No | Yes | 5 | Yes/TT×4 | No | 17 | - | CR |
| 8 | Male | No | 10 | CR | No | Yes | 5 | Yes/IT×4 | No | 7.5 | - | CR |
| 9 | Male | No | 10 | CR | No | Yes | 4 | No | Intracranial relapsed after 4 cycles of immunotherapy | 8 | 5 | DOD |
| 10 | Male | No | 10 | PR (MIBG score 5, lesions in the lateral aspect of the left orbit, left sphenoid bone, vertebral pedicles, and bilateral hip bones) | Yes | Yes | 3 | No | Sphenoid bone relapsed after 3 cycles of immunotherapy | 20 | 4 | After relapsed, re‑treatment achieved CR; the therapies included chemotherapy, radiotherapy, 3F8, and DFMO. |
| 11 | Male | Yes | 7 | CR | No | Yes | 5 | No | No | 42 | - | CR |
| 12 | Female | Yes | 10 | CR | No | Yes | 5 | Yes/IT×4 | DFMO (3 months after discontinuation of immunotherapy) | 23 | 14 | Eight months after discontinuation immunotherapy, and 4 months after oral administration of DFMO, relapsed occurred in the mediastinal and cervical lymph nodes, and the patient underwent re‑treatment. |
| 13 | Female | No | 8 | CR | No | Yes | 5 | Yes/IT×4 | No | 8 | - | CR |
| 14 | Male | Yes | 8 | CR | Yes | Yes | 6 | No | No | 7 | - | CR |

*ASCT, autologous stem cell transplantation; CR, complete response; DMFO, difluoromethylornithine; IT, irinotecan/temozolomide; MYCN, v-myc myelocytomatosis viral oncogene; RT, radiotherapy; TT, topotecan/temozolomide*

**Supplementary Table 2. Detail information of 14 patients with PR prior to dinutuximab beta**

| **No.** | **Sex** | **MYCN amplification** | **Cycles of induction chemotherapy** | **Disease status at the end of induction therapy** | **ASCT** | **RT** | **Cycles of dinutuximab beta** | **Combination Chemotherapy** | **MIBG/PET/CT scores prior immunotherapy** | **MIBG scores of BM minimal residual disease after 2 cycles immunotherapy** | **MIBG Scores after immunotherapy** | **Other treatments after immunotherapy** | **Follow-up duration (months)** | **Time of event onset (months)** | **Disease status** |
| --- | --- | --- | --- | --- | --- | --- | --- | --- | --- | --- | --- | --- | --- | --- | --- |
| 15 | Female | No | 15 | PR (BM minimal residual disease, MIBG score 10; multiple bone lesions, right diaphragmatic crus) | Yes | No | 6 | Yes/IT×3 | 3 (left humerus, bilateral ilia), BM minimal residual disease | Increased uptake in the right diaphragmatic crus on MIBG → abdominal and right diaphragmatic crus radiotherapy / BM minimal residual disease turned negative | 0 | DMFO (14 months after discontinuation of immunotherapy) | 33 | 30 | Retreatment after relapse (22 months after stopping immunotherapy and 8 months after oral DFMO, BM minimal residual disease turned positive; MIBG score 1 in the right femur) |
| 16 | Male | No | 10 | PR (MIBG-positive; skull base bones, vertebral bones, pelvic bones) | No | Yes | 6 | Yes/TT×3 | Suspicious MIBG score 2 (lateral wall of the right orbit, right ilium) | 0 | 0 | DMFO (2 months after discontinuation of immunotherapy) | 25 | - | CR |
| 17 | Female | No | 9 | PR (MIBG score 4; same as before antibody treatment) | No | Yes | 6 | Yes/IT×4 | 4 (retroperitoneal surgical field, left ilium, small lymph nodes at the left renal hilum) – all had received proton radiotherapy before antibody therapy | 0 | 0 | DMFO (10 months after discontinuation of immunotherapy) | 25 | - | CR |
| 18 | Male | No | 8 | PR (MIBG score 1; same as before immunotherapy) | No | Yes | 5 | No | 1 (left 2nd rib) – had received radiotherapy before antibody therapy | 0 | 0 | No | 12 | - | CR |
| 19 | Male | No | 9 | PR (MIBG score 10; paravertebral soft tissue of T6-T8, involving intervertebral foramen, skull, vertebral bodies, femurs, tibias, and other multiple bone lesions) | Yes | Yes | 6 | Yes/TT×2 | 9 (right 7th rib, paravertebral soft tissue of T6-T7, sacrum, retroperitoneum) – had received radiotherapy before antibody therapy | Score 4 + suspicious score 1 (T6-T7 vertebral bodies, right paravertebral soft tissue, right soft tissue of S1-S2, right 7th rib, L5 vertebral body, S1 vertebral body; uptake was lower than before) | 0 | DMFO (2 months after discontinuation of immunotherapy) | 12 | - | CR |
| 20 | Female | No | 10 | PR (MIBG score 5; T4 vertebral body, right side of sacrum, right ischium, lower segment of left femur, left ilium) | No | Yes | 6 | Yes/IT×4 | 1 (lower segment of the left femur) – had received radiotherapy before antibody therapy | 1 (Same as before) | 1 (MIBG Scores after immunotherapy: the findings were the same as those before treatment, but the uptake was significantly reduce) | DMFO (3 months after discontinuation of immunotherapy) | 23 | - | PR |
| 21 | Female | No | 11 | PR (MIBG score 3; cervical lymph nodes, mediastinal soft tissue, lateral wall of left orbit) | No | Yes | 6 | No | Suspicious score 1 (right femur) | - | 1 (increased uptake was observed in the middle and lower segments of the right femur, with local bone and soft tissue progression) | PD occurred 1 month after discontinuation of antibody therapy, followed by chemotherapy combined with radiotherapy | 30 | 8.5 | PD and retreatment |
| 22 | Male | Yes | 13 | PR (multiple bone and soft tissue lesions) | No | Yes | 5 | No | 21 (multiple bones, S3 sacral foramen, and soft tissues) |  | 21 (Same as before) | No | 44 | 24 | Retreatment after recurrence (1.5 years after discontinuation of immunotherapy; MIBG score 23; new onset or progression of lesions compared with previous findings, involving the sphenoid bone, left side of the clivus, left posterior roof of the nasopharynx, cervicothoracic vertebral bones and soft tissues, intraforaminal region, vertebral foramen, and BM minimal residual disease) |
| 23 | Male | Yes | 11 | PR (same as before immunotherapy) | No | Yes | 3 | Yes/TT×2 | 2 (lower segment of the right femur, mediastinal lymph nodes, anterior to the posterior mediastinal spine, soft tissue adjacent to the thoracic aorta) – all had received radiotherapy before immunotherapy | 2 (Same as before) | - | Naxitamab | 9 |  | Stable disease (after 3 cycles of immunotherapy the MIBG score remained unchanged; the parents requested switching to naxitamab; recent MIBG score 1 in the right femur) |
| 24 | Male | No | 8 | PR (same as before immunotherapy) | No | Yes | 5 | Yes/IT×4 | PET/CT (thoracolumbar spine) | - | PR (the uptake on PET/CT was significantly decreased) |  | 8 | - | PR |
| 25 | Male | No | 11 | PR (MIBG score 11; mediastinal lymph nodes, multiple bones, BM minimal residual disease) | No | Yes | 5 | Yes/TT×4 | 2 (bilateral ilia) | 0 | 0 | No | 7 | - | CR |
| 26. | Female | No | 9 | PR (same as before immunotherapy) | No | Yes | 2 | Yes/IT×1 | 2 (right humerus and ulna-radius), bone marrow MRD | 2 (Same as before)、BM minimal residual disease turned negative | - | Maintenance Therapy | 6 | 2.5 | Stable disease (discontinue the treatment due to financial reasons) |
| 27 | Female | No | 7 | PR | Yes | Yes | 5 | No | 1 (left side of the sacrum) (sacral radiotherapy was administered after the 2nd cycle of immunotherapy) | 0 | 0 | DMFO (3 months after discontinuation of immunotherapy) | 18 | - | CR |
| 28 | Male | No | 8 | PR | No | Yes | 5 | Yes/IT×4 | 1 (lower mediastinum adjacent to the thoracic aorta) – had received radiotherapy before immunotherapy | 0 | 0 | DMFO (4 months after discontinuation of immunotherapy) | 16 | - | CR |

*ASCT, autologous stem cell transplantation; BM, bone marrow; CR, complete response; DMFO, difluoromethylornithine; IT, irinotecan/temozolomide; MIBG, meta-iodobenzylguanidine; MYCN, v-myc myelocytomatosis viral oncogene; RT, radiotherapy; TT, topotecan/temozolomide; PET/CT, positron emission tomography/ computed tomography; PR, partial response*

**Supplementary Table 3. MIBG Curie score from baseline to post-treatment in 12 patients**

| **No.** | **Prior treatment** | **Cycle 2 or 3** | **Cycle 5 or 6 (EOT)** | **1-year follow-up** | **At the last of follow-up** |
| --- | --- | --- | --- | --- | --- |
| 15 | 3 (Bone+ BM) | 1 (Bone+ BM) | 0 | 0 | 1 (Bone+ BM) |
| 16 | 2 (Bone) | 0 | 0 | 0 | 0 |
| 17 | 4 (Bone+ soft tissue) | 0 | 0 | 0 | 0 |
| 18 | 1 (Bone) | 0 | 0 | 0 | 0 |
| 19 | 9 (Bone+ soft tissue) | 5 (Bone+ soft tissue) | 0 | 0 | 0 |
| 20 | 1 (Bone) | 1 (Bone) | 1 (Bone) | 1 (Bone) | 1 (Bone) |
| 21 | 1 (Bone) | 1 (Bone) | 1 (Bone) | 1 (Bone) | 14 (Bone) |
| 23 | 2 (Bone+ soft tissue) | 2 (Bone+ soft tissue) | 2 (Bone+ soft tissue) | 2 (Bone+ soft tissue) | 1 (Bone) |
| 25 | 2 (Bone) | 0 | 0 | 0 | 0 |
| 26 | 2 (Bone+ BM) | 2 (Bone+ BM) | 2 (Bone+ BM) | 2 (Bone+ BM) | 2 (Bone+ BM) |
| 27 | 1(Bone) | 0 | 0 | 0 | 0 |
| 28 | 1 (soft tissue) | 0 | 0 | 0 | 0 |

*EOT, end of treatment; BM, bone marrow; MIBG, meta-iodobenzylguanidine*

**Supplementary Table 4. Adverse events in 10 patients who received the dinutuximab beta without chemotherapy in the all cycles.**

|  | **Cycle 1 (n=10)** | | | **Cycle 2 (n=10)** | | | **Cycle 3 (n=10)** | | | **Cycle 4 (n=9)** | | | **Cycle 5 (n=8)** | | |
| --- | --- | --- | --- | --- | --- | --- | --- | --- | --- | --- | --- | --- | --- | --- | --- |
| **Grade** | **1 and 2** | **3** | **4** | **1 and 2** | **3** | **4** | **1 and 2** | **3** | **4** | **1 and 2** | **3** | **4** | **1 and 2** | **3** | **4** |
| Fever | 8(80%) | 2(20%) |  | 8(80%) | 1(10%) |  | 2(20%) |  |  |  |  |  | 2(25%) |  |  |
| Pain | 4(40%) |  |  | 2(20%) |  |  | 1(10%) |  |  |  |  |  |  |  |  |
| Hypotension | 2(20%) |  |  |  |  |  |  |  |  |  |  |  |  |  |  |
| Hypoxemia | 1(10%) |  |  |  |  |  |  |  |  |  |  |  |  |  |  |
| Capillary leak syndrome | 3(30%) |  |  |  |  |  |  |  |  |  |  |  |  |  |  |
| Rash | 4(40%) |  |  | 1(10%) |  |  | 1(10%) |  |  |  |  |  |  |  |  |
| Infection | 1(10%) | 1(10%) |  |  |  |  | 1(10%) |  |  | 1(11.1%) |  |  |  |  |  |
| Hypomyotonia |  |  |  | 1(10%) |  |  |  |  |  |  |  |  |  |  |  |
| Diarrhea | 5(50%) |  |  | 1(10%) |  |  | 1(10%) |  |  | 1(11.1%) |  |  |  |  |  |
| Chill | 5(50%) |  |  | 1(10%) |  |  |  |  |  |  |  |  |  |  |  |
| Cough | 4(40%) |  |  | 1(10%) |  |  |  |  |  |  |  |  |  |  |  |
| Vomit | 1(10%) |  |  |  |  |  |  |  |  |  |  |  |  |  |  |
| Eye abnormalities | 1(10%) |  |  | 3(30%) |  |  | 1(10%) |  |  | 1(11.1%) |  |  | 1(12.5%) |  |  |
| Anemia | 4(40%) | 1(10%) |  | 3(30%) |  |  | 1(10%) |  |  |  |  |  |  |  |  |
| Neutropenia | 1(10%) |  |  |  |  |  |  |  |  |  |  |  |  |  |  |
| Thrombocytopenia |  | 1(10%) |  | 1(10%) |  |  | 1(10%) |  |  | 1(11.1%) |  |  | 1(12.5%) |  |  |
| Low albumin | 9(90%) |  |  | 6(60%) |  |  | 1(10%) |  |  | 1(11.1%) |  |  |  |  |  |
| Elevate GGT | 5(50%) | 2(20%) |  | 2(20%) |  |  |  |  |  |  |  |  |  |  |  |
| Elevate ALT | 3(30%) | 1(10%) |  | 1(10%) |  |  |  |  |  |  |  |  |  |  |  |
| Prolonged APTT | 3(30%) |  |  | 1(10%) |  |  |  |  |  |  |  |  |  |  |  |
| Electrolyte imbalance | 3(30%) |  |  | 2(20%) |  |  |  |  |  |  |  |  |  |  |  |

*ALT, Alanine Aminotransferase; APTT, Activated Partial Thromboplastin Time; CGT, γ-Glutamyl Transferas*

**Supplementary Table 5. Adverse events in 18 patients who received the dinutuximab beta with chemotherapy after the first cycle.**

|  | **Cycle 2 (n=18)** | | | **Cycle 3 (n=17)** | | | **Cycle 4 (n=16)** | | | **Cycle 5 (n=16)** | | | **Cycle 6 (n=6)** | | |
| --- | --- | --- | --- | --- | --- | --- | --- | --- | --- | --- | --- | --- | --- | --- | --- |
| **Grade** | **1 and 2** | **3** | **4** | **1 and 2** | **3** | **4** | **1 and 2** | **3** | **4** | **1 and 2** | **3** | **4** | **1 and 2** | **3** | **4** |
| Fever | 13(72.2%) | 1(5.6%) | 1(5.6%) | 11(64.7%) |  |  | 7(43.8%) |  |  | 6(37.5%) |  |  | 1(16.7%) |  |  |
| Pain | 6(33.3%) |  |  | 1(5.9%) |  |  | 3(18.8%) |  |  |  |  |  |  |  |  |
| Hypoxemia | 1(5.6%) |  |  |  |  |  |  |  |  |  |  |  |  |  |  |
| Rash |  | 1(5.6%) |  | 3(17.6%) |  |  | 5(31.3%) |  |  | 1(6.3%) |  |  |  |  |  |
| Infection # | 2(11.1%) |  |  | 2(11.8%) | 2(11.8%) |  | 1(6.3%) |  |  | 1(6.3%) |  |  |  |  |  |
| Diarrhea | 8(44.4%) |  |  | 5(29.4%) |  |  | 6(37.5%) |  |  | 1(6.3%) |  |  |  |  |  |
| Ileus | 1(5.6%) |  |  |  |  |  |  |  |  |  |  |  |  |  |  |
| Chill | 1(5.6%) |  |  |  |  |  | 1(6.3%) |  |  |  |  |  |  |  |  |
| Cough | 4(22.2%) |  |  | 4(23.5%) |  |  | 5(31.3%) |  |  | 2(12.5%) |  |  |  |  |  |
| Conjunctivitis | 3(16.7%) |  |  | 2(11.8%) |  |  | 4(25.0%) |  |  | 4(25.0%） |  |  |  |  |  |
| Oral mucositis | 11(61.1%) |  |  | 1(5.9%) |  |  |  |  |  |  |  |  |  |  |  |
| Anemia | 12(66.7%) |  |  | 10(58.9%) |  |  | 5(31.3%) |  |  | 3(18.8%) | 1(6.3%) |  | 1(16.7%) |  |  |
| Neutropenia | 12(66.7%) | 4(22.2%) | 1(5.6%) | 3(17.6%) | 3(17.6%) |  | 3(18.8%) | 1(6.3%) |  |  | 2(12.5%) | 1(6.3%) |  |  |  |
| Thrombocytopenia | 6(33.3%) | 5(27.8%) | 1(5.6%) | 4(23.5%) | 2(11.8%) |  | 3(18.8%) | 2(12.5%) |  | 3(18.8%) | 4(25.0%) | 1(6.3%) |  |  |  |
| Low albumin | 15(83.3%) |  |  | 11(64.7%) |  |  | 8(50.0%) |  |  | 7(43.8%) |  |  |  |  |  |
| Elevate GGT | 5(27.8%) |  |  | 2(11.8%) |  |  | 2(12.5%) |  |  | 1(6.3%) |  |  |  |  |  |
| Elevate ALT | 4(22.2%) |  |  | 2(11.8%) |  |  | 1(6.3%) |  |  | 1(6.3%) |  |  |  |  |  |
| Prolonged APTT | 6(33.3%) |  |  | 4(23.5%) |  |  | 1(6.3%) |  |  | 2(12.5%) |  |  |  |  |  |
| Arrhythmian | 1(5.6%) |  |  |  |  |  |  |  |  |  |  |  |  |  |  |
| Electrolyte imbalance | 4(22.2%) |  |  | 3(17.6%) |  |  | 1(6.3%) |  |  |  |  |  |  |  |  |

*ALT, Alanine Aminotransferase; APTT, Activated Partial Thromboplastin Time; CGT, γ-Glutamyl Transferas*
